# Mechanisms of heme transport in the mitochondria

**DOI:** 10.1042/BST20253013

**Published:** 2025-05-29

**Authors:** Saieeda Fabia Ali, Adrianna E. White, Amy Medlock, Oleh Khalimonchuk

**Affiliations:** 1 Department of Biochemistry, University of Nebraska-Lincoln, Lincoln NE 68588U.S.A.; 2 Department of Biochemistry and Molecular Biology, University of Georgia, Athens GA 30602U.S.A.; 3 Department of Interdisciplinary Biomedical Sciences, University of Georgia, Athens GA 30602U.S.A.; 4 Nebraska Redox Biology Center, Lincoln NE 68588U.S.A.; 5 Fred & Pamela Buffett Cancer Center, Omaha NE 68198U.S.A.

**Keywords:** heme, heme biosynthesis, hemoproteins, membrane transporters, mitochondria

## Abstract

Heme is a vital but highly reactive compound that is synthesized in mitochondria and subsequently distributed to a variety of subcellular compartments for utilization. The transport of heme is essential for normal cellular metabolism, growth, and development. Despite the vital importance of heme transport within the cell, data are lacking about how newly synthesized heme is shuttled within the mitochondrion or exported from the organelle. Here, we briefly summarize current knowledge about the process of mitochondrial heme distribution and discuss the current unresolved questions pertinent to this process.

## Introduction

Heme is a metal-containing hydrophobic organic compound, composed of a porphyrin ring with a central iron, either ferrous (Fe^2+^) or ferric (Fe^3+^), coordinated by the nitrogen atoms of the porphyrin. As a prosthetic group in proteins, heme is essential for a plethora of biological functions [1,2]. In eukaryotic cells, it plays crucial roles in gas sensing, oxygen transport and binding, NO synthesis, and electron transfer [3–6]. Additionally, heme has been shown to interact with several transcription factors and microRNA processing proteins, thus regulating gene expression. Through these activities, heme is involved in controlling a number of physiological functions, including iron homeostasis, mitochondrial respiration, mitophagy, apoptosis, circadian rhythm, cell proliferation, and stress responses [7–10]. Paradoxically, heme is highly reactive and inherently toxic in its free form, owing to its redox-active nature. Free or labile heme can intercalate into biological membranes due to its hydrophobicity and non-specifically bind to proteins. In its free form, heme can produce hydroxyl radicals, resulting in oxidative damage to cellular components [11–13]. Furthermore, heme biosynthetic intermediates are also reactive; thus, imbalances in heme synthesis, which result in substrate accumulation, can cause cellular and tissue damage, as observed in the porphyrias [14]. Therefore, heme biosynthesis and distribution necessitate tight control of the concentration and availability of heme and its intermediates, as well as the monitoring of import, synthesis, degradation, and export levels thereof.

**Figure 1:**
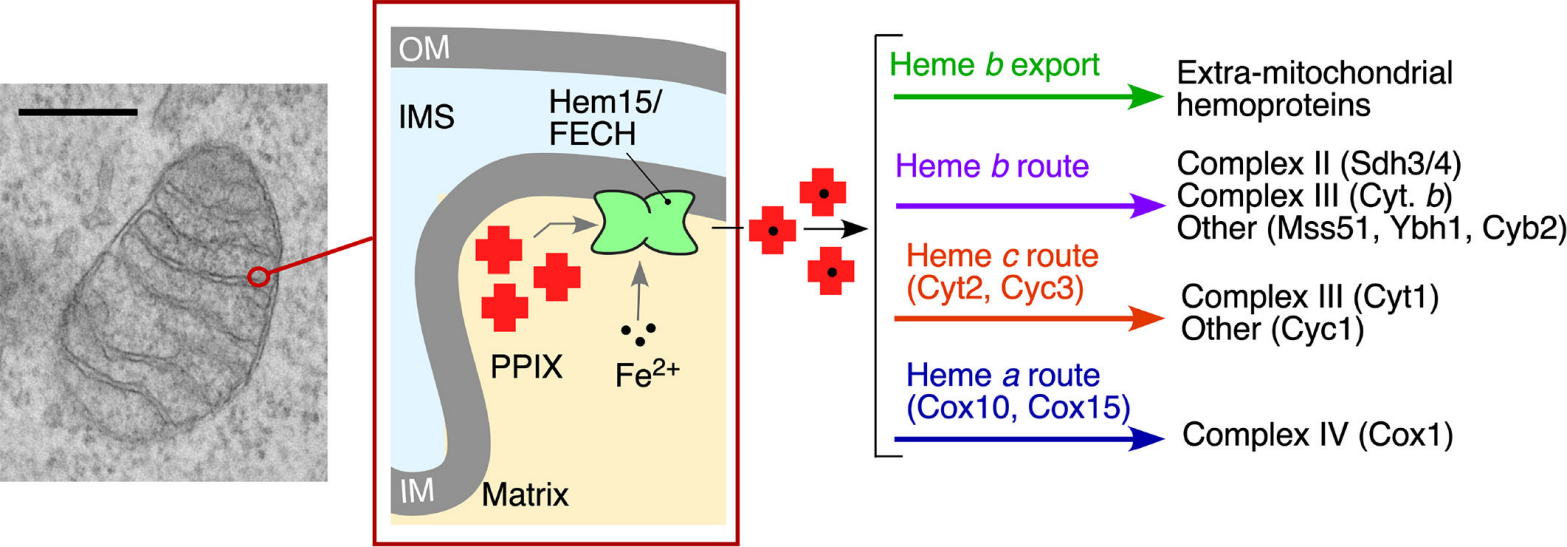
Overview of the mitochondrial heme routes. Following heme synthesis in the mitochondrial matrix, heme must be transported to hemoproteins throughout the cell. Proposed routes and proteins are shown and described in the review. A representative transmission electron microscopy micrograph of mitochondria (scale bar, 0.4 µm) from wildtype mouse embryonic fibroblasts is used to highlight the location of subcompartments where the final step of heme biosynthesis is taking place. Hem15/FECH, ferrochelatase; IM, inner mitochondrial membrane; IMS, intermembrane space; OM, outer mitochondrial membrane; PPIX, protoporphyrin IX.

Heme synthesis occurs in virtually all eukaryotic organisms except for certain parasites and nematodes [15–17]. For example, the roundworm *Caenorhabditis elegans* does not synthesize heme and thus acquires it through its diet [18]. The canonical heme synthetic pathway consists of eight steps that take place in both mitochondria and cytosol and has been recently reviewed [19]. One unique feature of the eukaryotic heme biosynthetic pathway is the compartmentalization of its initial and final steps to the mitochondria. The terminal step in heme biosynthesis is mediated by the enzyme ferrochelatase (FECH) that incorporates an iron atom into the protoporphyrin IX to produce protoheme [20], a *b*-type heme, which is a predominant heme species in cells. Subsequently, heme is rapidly and safely moved to multiple sites for utilization (Figure 1). Whereas some heme distribution routes, such as those leading to the formation of mitochondria-specific *c*- and *a*-type hemes and subsequent hemylation of cytochrome *c* and cytochrome *c* oxidase (CcO), respectively, are fairly well understood [5,21], others, including the pathways and proteins necessary for the distribution of heme *b* within and outside of the mitochondria, remain largely elusive. In this review, we will briefly summarize current knowledge about the process of mitochondrial heme transport, with a specific emphasis on the key factors involved in heme bioavailability. In addition, we will highlight the physiological significance of mitochondrial heme trafficking in different cellular environments and its implications in disease states. Finally, we will discuss the currently unresolved questions in the field and potential directions for future research.

## Heme distribution

Following heme synthesis, the newly made heme *b* needs to be securely routed, with a significant portion of the compound directed toward export from the mitochondrion. Using a series of genetically encoded radiometric sensors for labile heme that were targeted to various subcellular compartments, it was established that the steady-state levels of labile heme are quite different between various cellular subcompartments: ~20–40 nM in the cytosol and ~2 nM in the nucleus and mitochondrial matrix [1]. Interestingly, trafficking of labile heme into the mitochondrial matrix and cytosol occurred at similar rates, while trafficking to the nucleus is 25% faster [22]. These findings suggested that once heme is synthesized at the matrix side of the inner mitochondrial membrane (IMM), it is dispersed to multiple compartments by parallel pathways. Although the molecular basis of such rapid distribution remains largely elusive, it is clear that the process of heme export is remarkably efficient and well orchestrated. Indeed, unlike the cytosol and the nucleus, wherein the surplus heme could be eliminated by heme oxygenase HO-1 [12,23,24], mitochondria do not harbor such an enzyme and rely on intricate regulation of the heme biosynthetic pathway and efficient heme export mechanisms. However, both the regulation of mitochondrial heme biosynthesis and its trafficking remain incompletely understood.

Underscoring the importance of heme management in the mitochondria, heme is a prosthetic group that is crucial for multiple mitochondrial pathways and reactions, including the Krebs cycle, oxidative metabolism [25,26], and mitochondrial cytochromes P450 (CYP450) enzymes that are required for steroidogenesis, biosynthesis for bile acids, and 1,25-dihydroxyvitamin D_3_ [27]. Malfunctions of this system have been linked to a number of clinical conditions, including lung injury, neurodegeneration, and cardiac dysfunction [11,28], further highlighting the physiological importance of heme homeostasis. The following sections will briefly survey our current understanding of the factors implicated in mitochondrial heme export.

Whereas the chemical aspects of heme biosynthetic reactions are well understood, the trafficking of newly synthesized heme and its subsequent delivery to the target proteins remain far from clear. In fact, remarkably little is known about how heme is transported within the mitochondria following its synthesis. A number of proteins have been proposed to play roles in heme homeostasis based solely on their ability to bind heme *in vitro*, with little or no experimental evidence of their role in heme-dependent processes *in vivo* or *in cellulo* [29,30]. Such results need to be carefully analyzed, as mere heme binding may not always reflect a protein’s physiological role. For example, the flavoprotein Sdh1—an enzymatic subunit of succinate dehydrogenase (complex II)— was shown to bind to hemin-agarose beads through its FAD-binding domain [31]. Similarly, the two purported cell surface heme importers—the heme carrier protein HCP1 and feline leukemia virus subgroup C receptor FLVCR2—that were identified in a similar fashion, likely do not play roles in physiological heme transport [5]. Indeed, HCP1 was later shown to be a proton-coupled folate transporter, and FLVCR2 has not been demonstrated to directly transport heme, and its overexpression sensitizes cells to heme toxicity only at very high (~200 μM) heme concentrations [5]. Contrarily, evidence exists to support several mitochondria-related factors and pathways that have been found to both bind and buffer intracellular heme, and/or regulate heme bioavailability in different cells as described herein.

## The heme *a* route

**Figure 2:**
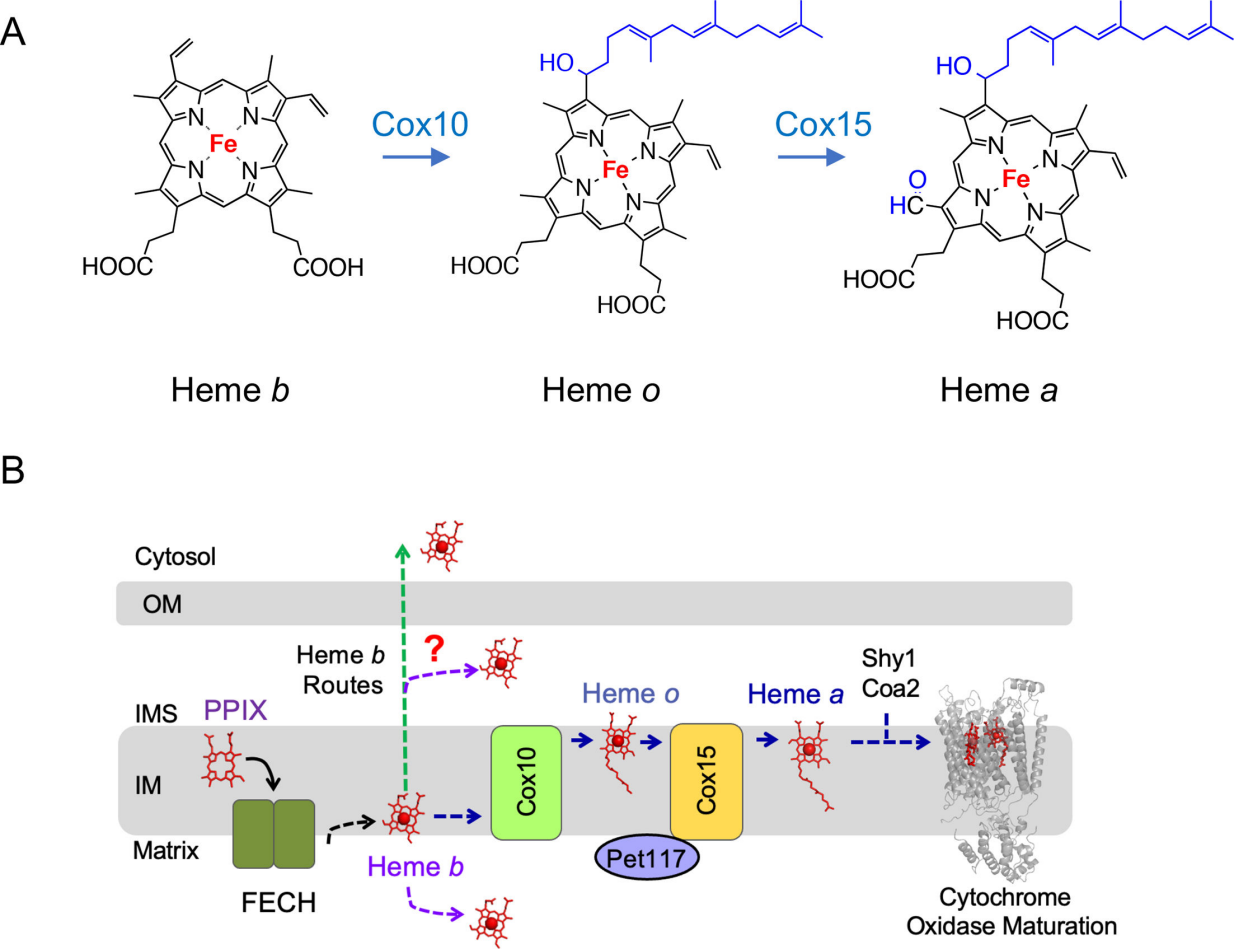
The heme *a* route. (**A**) Chemical structures of the heme species in the heme *a* pathway. Heme *a* is produced from the heme *b* (also known as protoheme) precursor in a two-step reaction mediated by Cox10 (heme *o* synthase) and Cox15 (heme *a* synthase) enzymes. The reaction involves the formation of a short-lived heme *o* intermediate. (**B**) Mitochondrial heme *b* distribution and heme *a* route. Ferrochelatase (FECH) metalates protoporphyrin IX (PPIX), yielding heme *b*, which is rapidly routed to subcellular locations via an unknown mechanism. In the heme *a* pathway, heme *b* is trafficked via Cox10 and Cox15, which modify it to heme *a*. Heme *a* is inserted into maturing CcO by assembly factors Coa2 and Shy1. The assembly factor Pet117 appears to be also involved in this process. IM, inner mitochondrial membrane; IMS, intermembrane space; OM, outer mitochondrial membrane.

One major route for the newly synthesized heme *b* is the heme *a* biosynthetic route. Heme *a* is a modified heme *b* that is uniquely used by CcO, a key heme-copper enzyme of the mitochondrial respiratory chain [21,32]. The two identical heme *a* molecules, albeit with different coordination geometries—designated *a* and *a*_3_—reside in the Cox1 core subunit of CcO and are essential for catalysis and the stability and folding of Cox1 [21,32]. Heme *a* is generated through a two-step conversion of heme *b* mediated by the evolutionarily conserved enzymes, Cox10 and Cox15 (Figure 2A and B). The former enzyme mediates farnesylation of the 2-position vinyl group of heme b and yields a heme *o* intermediate, which is then converted to heme *a* by Cox15-mediated oxidation of the C8 pyrrole methyl moiety [21,33] (Figure 2A). Cox15-catalyzed conversion of heme *o* to heme *a* occurs in conjunction with ferredoxin and ferredoxin reductase [34–36]. While the directionality of this route is clear, many of its aspects remain enigmatic. For example, it remains unclear how heme *b* is transferred from FECH to Cox10. Similarly, although intuitive, the transfer of heme *o* intermediate between Cox10 and Cox15 remains to be demonstrated. Cox10 and Cox15 are large polytopic proteins with limited structural information available for either protein [21,33]. They are differentially regulated, do not appear to form a stable complex, and Cox15 is present in ~8-fold excess over Cox10 [37], thus adding more intrigue to the question of how heme is transferred via this axis. Studies in yeast established that at least two additional molecules—Shy1/SURF1 and Coa2, which do not have clear orthologs in higher eukaryotes—play an important role in heme *a* delivery to Cox1 [21,38]. The formation of the heme *a* and heme *a_3_* centers appears to occur within the Shy1-containing Cox1 assembly intermediate [21,39], wherein Shy1 appears to chaperone maturation of the heme *a_3_* site rather than serve as a direct heme *a* donor to the newly synthesized Cox1. Several reports suggested that Cox15 may associate with Shy1 and complex III–IV assembly intermediates, but the significance of these associations remains to be investigated [40,41]. Coa2 is a small membrane-associated protein that co-operates with Shy1 in Cox1 hemylation [38,42,43]. Cells lacking Coa2 exhibit rapid degradation of the newly synthesized Cox1, arising from impaired hemylation and the subsequent misfolding of the molecule [38,42]. The lack of Coa2 can be compensated for by the deletion of the mitochondrial peptidase Oma1 [38,42], the dominant gain-of-function N196K Cox10 mutation [38], or by simultaneous increase in the Cox1 synthesis and levels of wildtype Cox10 [42]. Additionally, a conserved CcO assembly factor Pet117 appears to be important for stabilization of Cox15 oligomers and Cox1 hemylation [44]. Further studies are warranted to elucidate the details of this pathway.

## The heme *c* route

**Figure 3:**
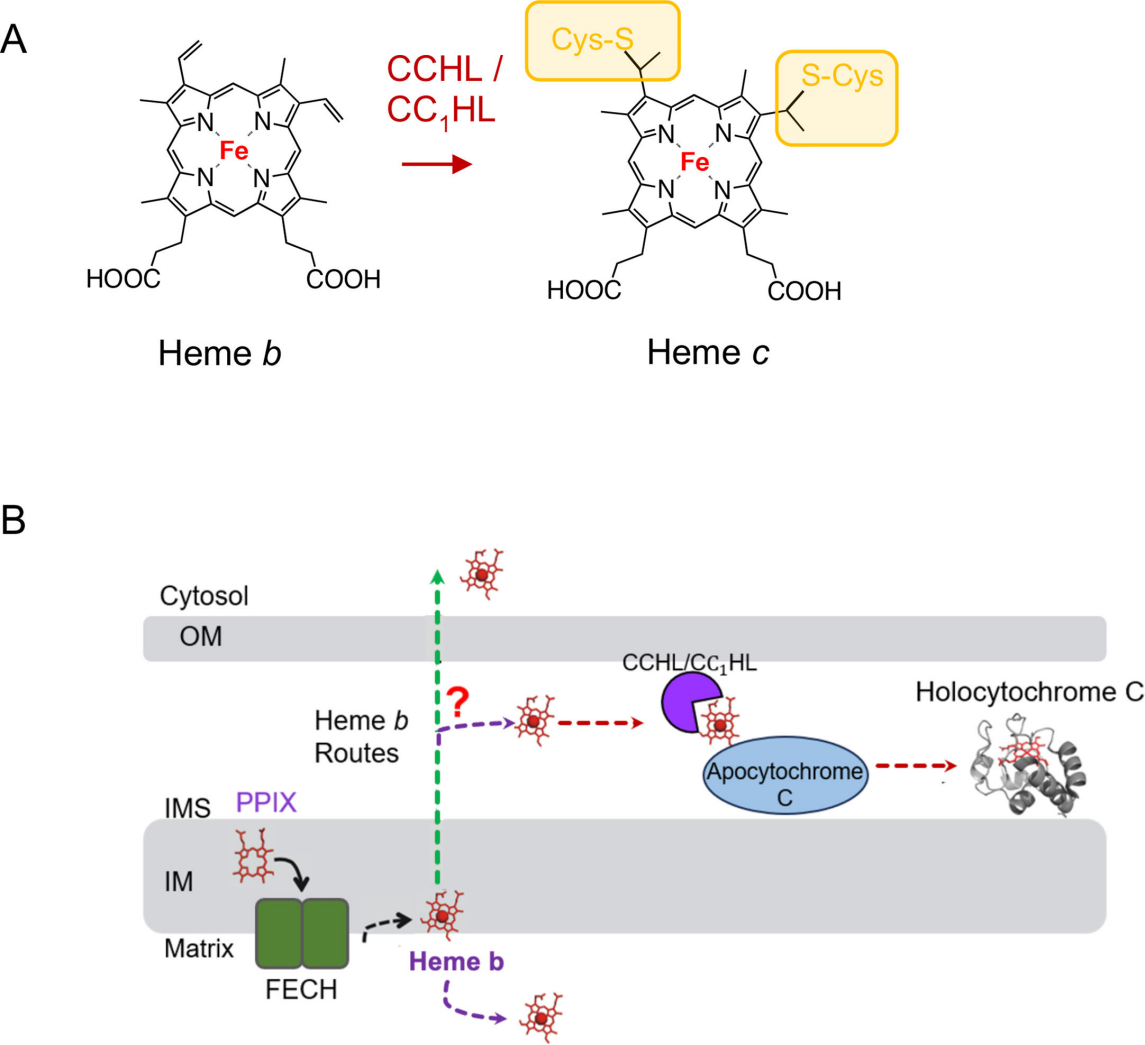
The heme *c* route. (**A**) Chemical structures of the heme species in the heme *c* pathway. Heme *c* is generated from heme *b* through a cytochrome *c*/*c_1_* heme lyase-mediated covalent attachment to the sulfhydryl moieties of its target proteins. (**B**) Mitochondrial heme *c* route. The newly synthesized heme *b* is routed to either of the CCHL enzymes that transfer it to its cytochromes. IM, inner mitochondrial membrane; IMS, intermembrane space; OM, outer mitochondrial membrane.

The heme *c* route is another heme distribution pathway, wherein heme *b* is routed and covalently attached to its client mitochondrial cytochromes: cytochrome *c* and complex III subunit called cytochrome *c*_1_ (Figure 3A and B). In either case, the two vinyl groups of heme *b* form covalent bonds with the protein cysteines of the conserved Cx_2_CH motif, and the histidine residue acts as an axial ligand for the heme iron atom (Figure 3A) [45,46]. The pathway is relatively well characterized *in vitro* and involves two intermembrane space (IMS)-residing heme lyases, Cyc3/cytochrome c heme lyase (CCHL) and Cyt2/CC_1_HL in yeast and a single holocytochrome *c* synthase (HCCS) in mammals [46,47]. In each case, an IMM-associated lyase binds a reduced heme *b* with subsequent binding of apocytochrome and formation of covalent thioester bonds within the lyase-ferrous heme-apocytochrome complex [48]. Once the covalent thioester bonds are formed, the hemylated cytochrome is released from the complex. The release appears to be facilitated by conformational distortion of the covalently bound heme molecule and its axial ligand-mediated decrease in heme-CCHL adducts [48,49]. Some other proteins, such as yeast-specific l-lactate cytochrome *c* oxidoreductase Cyb2, are likely to follow the same hemylation mechanism. It is possible that additional factors may aid this process *in vivo*. For example, in yeast, an IMS-localized flavoprotein Cyc2, though not conserved and dispensable for CCHL function, was reported to act as a heme reductase and/or redox regulator of heme lyase function [50,51]. One important facet of the heme *c* route remains a mystery, however. That is, how newly synthesized heme *b* is routed across the IMM and delivered to CCHL enzymes. This important question remains to be addressed.

## Other routes

Additional, though much less characterized, routes for heme *b* include pathways that lead to several mitochondrial enzymes, such as respiratory chain complexes succinate dehydrogenase (complex II) and ubiquinol-cytochrome *c* reductase (complex III), as well as IMS-localized CcO assembly factor COA7 and mitochondrial matrix-localized flavohemoglobin Yhb1/Neuroglobin [5,21,52]. Studies in yeast identified additional mitochondrial hemoproteins with no clear orthologs in higher eukaryotes. These include CcO assembly factor Mss51 and cytochrome *c* peroxidase Ccp1 [32,53]. In each case, very little is known as to how heme is delivered to these target proteins. Available knowledge on complex III biogenesis indicates that the formation of heme *b* redox sites within the complex is an early post-translational event that likely ensures an additional feedback regulatory mechanism to minimize the presence of unincorporated heme molecules and reduce negative effects thereof [54,55].

**Figure 4:**
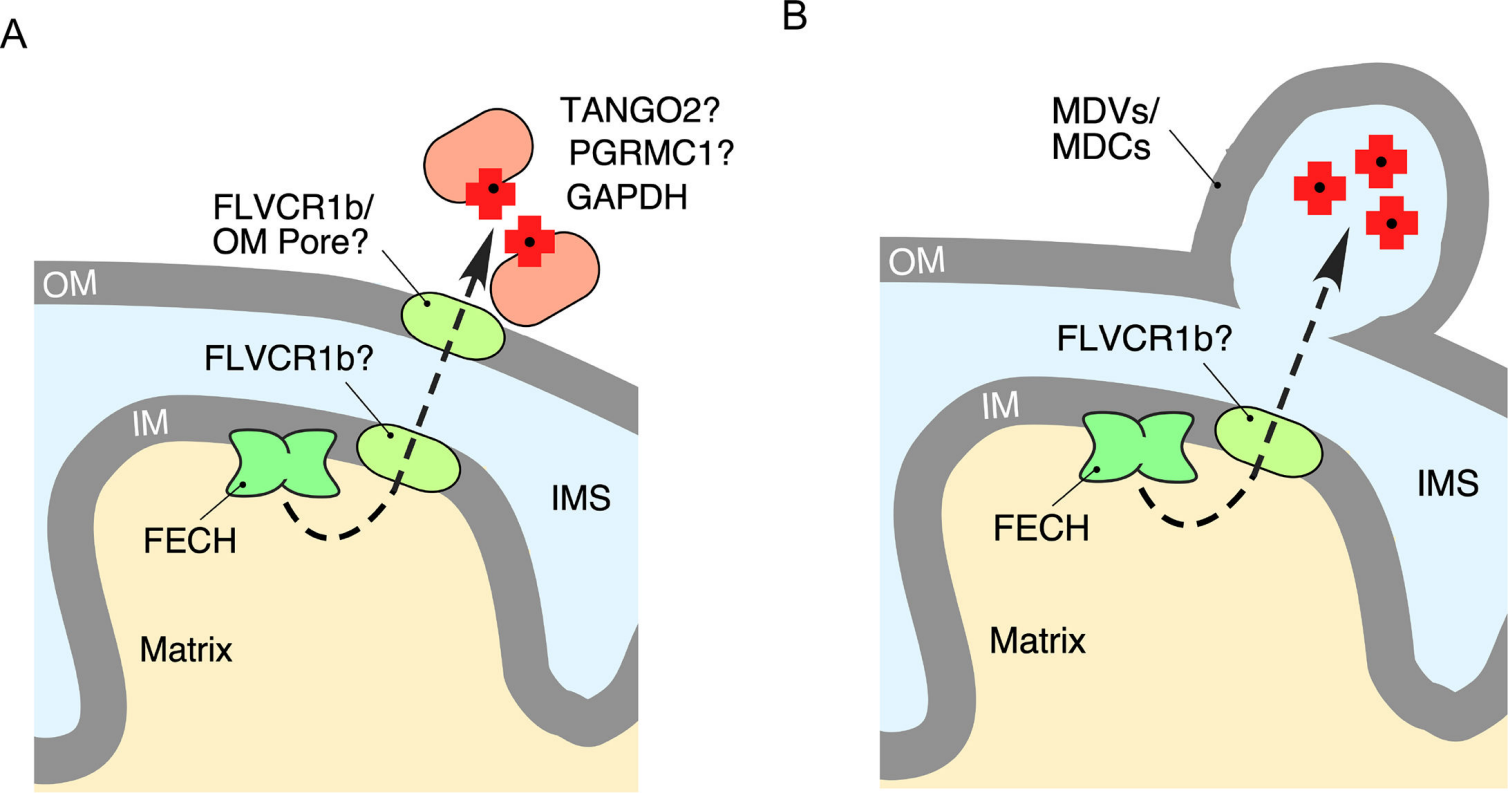
Models of mitochondrial heme export. (**A**) In the first model, the newly synthesized heme *b* is routed and exported from mitochondria via a dedicated transporter such as FLVCR1b. The exact localization of FLVCR1b remains to be investigated. (**B**) The second model posits that mitochondrial heme *b* is distributed via mitochondria-derived structures called mitochondria-derived vesicles or similar, yet distinct, mitochondria-derived compartments that are subsequently directed to endosomes and lysosomes. FECH, ferrochelatase; IM, inner mitochondrial membrane; IMS, intermembrane space; MDVs, mitochondria-derived vesicles; MDCs, mitochondria-derived compartments; OM, outer mitochondrial membrane.

Finally, an important route that remains far from clear is the export route. Indeed, a large portion of the mitochondria-produced heme must be mobilized and efficiently delivered to a variety of hemoproteins in other cellular compartments. Whereas it is generally believed that heme export could be arranged through a coordinated action of transporters (Figure 4A), the exact nature of said transporters remains elusive. One candidate transporter is an isoform of Feline leukemia virus subgroup C receptor-related protein FLVCR1, specifically FLVCR1b [56,57]. FLVCR1b is a shortened isoform that has six transmembrane domains. It is formed from an alternative transcription start site and appears to be mitochondria-localized [56,58]. Whereas FLVCR1 (sometimes referred to as FLVCR1a) functions as an exporter of cytosolic labile heme pool, controlling cell metabolism and oxidative status, the FLVCR1b variant was proposed to transport heme out of the mitochondria [56]. In cell culture models, it has been shown that overexpression of FLVCR1b increases cytosolic heme and that knockdown results in mitochondrial heme retention [56]. Recently, interactions between FLVCR1b and cytosolic heme chaperone glyceraldehyde phosphate dehydrogenase have been shown to trigger heme transfer between proteins and to downstream hemoproteins [59]. Even with these findings, questions about FLVCR1b still exist; in particular, the exact location of FLVCR1b in mitochondria and its alignment with heme biosynthetic machinery remain unclear, thus warranting further investigation.

The proteins progesterone receptor membrane component 1 (PGRMC1), its yeast homolog damage response protein 1, and its paralog progesterone receptor membrane component 2 (PGRMC2) are the factors implicated in heme trafficking. These proteins, along with two others in the membrane associate progesterone receptor family, Neudesin and Neuferricin, all share a cytochrome *b*_5_-like (Cyt *b*_5_) domain and all bind heme [60]. A notable difference found for the proteins in this family is that they have variable N-terminal domains. PGRMC1 and PGRMC2 both have an N-terminal extension that is proposed to be a single helical transmembrane domain, while Neudesin and Neuferricin are soluble proteins [61]. With respect to heme binding, all members of the family are thought to bind heme via a conserved Tyr (Tyr-113 in PGRMC1) found within the Cyt *b*_5_-like domain [62]. Crystallization of the soluble Cyt *b*_5_-like domain of PGRMC1 with heme bound reveals a solvent-exposed heme that dimerizes via heme stacking [62]. Moreover, PGRMC1 binds more tightly to ferric heme than to ferrous, consistent with tyrosinate coordination [63]. The interaction of PGRMC1 with the enzyme catalyzing the terminal step of heme synthesis, as well as its moderate affinity for heme, is consistent with this protein being involved in heme transfer [64]. Interestingly, carbon monoxide (CO) disrupts the formation of the PGRMC1 dimer, which is thought to be the active form [62]. This finding suggests that CO, which is produced as a byproduct of heme degradation, may be a feedback regulator of this protein. Biochemical evidence also supports PGRMC1 interacting with multiple CYP450 enzymes that regulate cholesterol, xenobiotic, bile acid, steroid, and arachidonic acid metabolism [60]. More recently, PGRMC2 was shown to function as a heme chaperone moving heme to the nucleus in brown adipose tissue to control mitochondrial function [65]. While PGRMC2 has been localized to the ER membrane and the nuclear envelope [66], PGRMC1 has reported localization throughout the cell [60]. Studies in a developing red blood cell model support that PGRMC1 is associated with the external face of the outer mitochondrial membrane [64], while other studies have found that it is associated with the ER membrane [67]. A more likely scenario is that PGRMC1 would localize to mitochondria-associated ER membranes, subcellular structures known to mediate ions, lipids, and metabolite exchange between the ER and mitochondrial networks [68].

Several recent studies reported that the mitochondrial heme biosynthetic enzymes, 5-aminolevulinate synthase and protoporphyrin oxidase, along with mitochondrial iron importer, mitoferrin, interact with FECH in a heme biosynthetic complex or metabolon [69,70]. This superstructure may therefore facilitate heme synthesis and distribution. Conceptually, this is not unprecedented and may resemble a recently reported coenzyme Q biosynthetic metabolon that encompasses virtually all of the enzymes involved in the mitochondrial ubiquinol biosynthesis [71]. In line with the above ideas, we recently showed that FECH is associated with mitochondrial intermembrane contacts via mitochondrial contact site and cristae organizing system machinery to facilitate the process of heme biosynthesis, especially when cells are forced to produce heme [72]. Likewise, as noted above, FECH has been shown to interact with PGRMC1 and PGRM2C [64,69,73], as well as a variety of other proteins in a cell-line-specific manner. Studies are underway to further explore the role, composition, and molecular details of the mitochondrial heme metabolon.

Yet another protein proposed to be involved in heme export is Transport and Golgi organization 2 (TANGO2). TANGO2 lacks transmembrane domains and is speculated to be localized in the cytoplasm, the Golgi, and the mitochondria [74]. Originally discovered in *Drosophila*, TANGO2 was proposed to be involved in heme trafficking based on the finding that mammalian and yeast cells deleted for this factor accumulate heme in mitochondria [75,76]. In *C. elegans,* TANGO2 homolog HRG-9 and its paralog HRG-10 were reported to be responsible for heme transport from intestinal cells [75]. Recent work supports interactions between TANGO2 and FLVCR1b possibly to support its export function [59], although other reports have challenged the protein’s heme transporting role [77–79]. As such, its role in mitochondrial heme export remains unclear.

Considering that heme is a hydrophobic lipid-like molecule that can intercalate in membranes, one alternative model for heme export and distribution would involve the mitochondrial membranes and cellular endomembranes. Indeed, interorganellar contact sites, such as mitochondria-ER contacts or mitochondria-vacuole/lysosome contacts were proposed to facilitate metabolites and lipid exchange between the organelles [80,81]. Similarly, the mitochondria-derived vesicles [82,83] or related mitochondria-derived compartments [84,85] were shown to encompass mitochondrial membranes and carry mitochondria-borne cargo, ultimately delivering to the endosomes and vacuole/lysosome. It is plausible that newly synthesized heme molecules could also be trafficked in such fashion (Figure 4B). In line with this notion, screens with genetically encoded heme sensors identified membrane remodeling GTPases as important players in mitochondrial heme distribution [22]. Studies are warranted to explore these emerging models.

## Disorders associated with dysfunctional mitochondrial heme transport

As noted herein, heme is an essential cofactor and signaling molecule. Therefore, it is not surprising that defects in mitochondrial heme synthesis and management lead to a number of human disorders, including anemias, porphyrias [86], and cardiovascular diseases [87–89]. With regard to the routes of distribution described in this review, the Mendelian mutations in heme *c* lyase HCCS have been shown to result in both defective cytochrome *c* hemylation and its apoptotic release. These variants manifest clinically as a hereditary dominant microphthalmia with linear skin defects (MLS) syndrome [45,90]. Similarly, hereditary mutations in conserved residues of heme *a* pathway enzymes COX10, COX15, and SURF1 result in a spectrum of severe disorders, including tubulopathy and leukodystrophy, sensorineural deafness, fatal infantile hypertrophic cardiomyopathy, Charcot-Marie-Tooth disease type 1A, and neurologic Leigh syndrome [87,88,91–102].

Mutations in FLVCR1 variants have been linked to posterior column ataxia and retinitis pigmentosa and Diamond-Blackfan syndrome in humans [103,104]. Additionally, FLVCR1 dysregulation has been associated with increased risk of diabetes and certain types of cancer [105,106]. Studies in the mouse models have shown that FLVCR1 deletion is associated with increased incidence of embryonic and *in utero* lethality and compromised erythropoiesis, resulting in hemorrhage and anemia in postnatal animals [10,107]. The FLVCR-deleted animals also present with skeletal malformations, dysfunctional muscle, and sensory neurodegeneration [107,108]. However, it remains to be determined which portion of these manifestations can be ascribed to defects in FLVCR1b variant.

Finally, TANGO2 deficiency has been associated with multiple pathologies including developmental delay, muscle weakness, intellectual delay, seizure, encephalopathy, arrhythmias, rhabdomyolysis, and increased chances of early-onset mortality in humans [109–111], underscoring its physiological importance. Given the multifaceted nature of this protein, studies are warranted to determine if and how defective heme metabolism contributes to these manifestations.

## Concluding remarks

Decades of research have led to a detailed understanding of the chemical aspects of the synthesis of heme; however, the mechanisms by which the newly synthesized heme is mobilized from FECH and routed within and exported outside of the mitochondria, and how the said processes are orchestrated and regulated are only beginning to emerge. This knowledge gap is in part due to our limited ability to monitor spatiotemporal dynamics of heme distribution and its subsequent conversions within the cell. Recent development of new tools, such as genetically encoded fluorescent heme sensors [29], has greatly supplemented traditional biochemical studies and has allowed us to overcome some of these technical challenges. Delineating how newly synthesized heme is routed within and exported from mitochondria and identifying factors that control this pathway will address a fundamental biological question and facilitate the development of new therapeutic approaches to treat patients with prevalent defects in heme management.

**Perspectives**

- Heme is a vital but highly reactive metal-containing hydrophobic compound synthesized in mitochondria that is important for many cellular functions within and beyond the mitochondria. Abnormalities in heme synthesis and distribution contribute to multiple prevalent pathological states, including anemias, porphyrias, and cardiovascular diseases.
- Some of the heme routes, such as heme a and heme c route, are well understood, whereas the others are only beginning to emerge. One major route is the heme export from mitochondria, for which several recently identified factors and models are discussed.
- Future studies will illuminate all pathways for heme distribution within and beyond the mitochondria and provide necessary information to better understand conditions that result in defects in mitochondrial heme synthesis and distribution.

## Abbreviations

CO: carbon monoxide
CYP450: cytochrome P450
CcO: cytochrome c oxidase
Cyt b_5_: cytochrome b_5_
FECH: Ferrochelatase
IMM: inner mitochondrial membrane
IMS: intermembrane space
PGRMC1: proteins progesterone receptor membrane component 1
PGRMC2: paralog progesterone receptor membrane component 2
TANGO2: Transport and Golgi organization 2
